# Attraction of phlebotomine sandflies to volatiles from skin odors of individuals residing in an endemic area of tegumentary leishmaniasis

**DOI:** 10.1371/journal.pone.0203989

**Published:** 2018-09-24

**Authors:** Diva da Silva Tavares, Vanessa Riesz Salgado, José Carlos Miranda, Paulo R. R. Mesquita, Frederico de Medeiros Rodrigues, Manoel Barral-Netto, Jailson Bittencourt de Andrade, Aldina Barral

**Affiliations:** 1 Instituto Gonçalo Moniz—Fiocruz–Salvador, Bahia—Brazil; 2 Faculdade de Medicina da Universidade Federal da Bahia (UFBA)–Salvador, Bahia–Brazil; 3 Faculdade de Medicina do Centro Universitário Christus (UNICHRISTUS)–Fortaleza, Ceará –Brazil; 4 Instituto de Química da Universidade Federal da Bahia (UFBA)–Salvador, Bahia–Brazil; 5 Instituto Nacional de Ciência e Tecnologia de Investigação em Imunologia (iii-INCT)–Salvador, Bahia–Brazil; 6 Instituto Nacional de Ciência e Tecnologia em Energia e Ambiente (INCT-EA)–Salvador, Bahia–Brazil; University of Richmond, UNITED STATES

## Abstract

**Background:**

Many studies have investigated what could attract insects of medical importance and a crucial role has lately been attributed to human skin odors. Most of these researches have been concerned with mosquitoes, e.g., vectors of dengue and malaria. Little is known about volatile organic compounds (VOCs) from human skin odors and their effects on leishmania vectors.

**Objective:**

The present study aimed to identify the VOCs from human skin that can be attractive to female anthropophilic phlebotomine sandflies.

**Results:**

Forty-two VOCs were identified from skin odors of 33 male volunteers, seven of which were tested in wind tunnel assays employing field-captured phlebotomine sandflies (75.4% identified as *Lutzomyia intermedia*). Hexane and (E)-oct-3-en-1-ol (octenol) were used as negative and positive controls, respectively. 2-Phenylacetaldehyde (hereafter called phenylacetaldehyde), 6-methylhept-5-en-2-one (also known as sulcatone), nonadecane and icosane were found to activate female phlebotomine sandflies, but only phenylacetaldehyde, 6-methylhepten-5-en-2-one and icosane elicited attraction responses.

**Conclusions:**

These results suggest that phenylacetaldehyde, 6-methylhepten-5-en-2-one and icosane may be suitable candidates for attractiveness experimentation in the field which can be an important tool to develop strategies concerning human beings protection against phlebotomine sandflies bites and consequently against leishmaniasis.

## Background

Knowledge concerning the chemical-ecological relations between phlebotomine sandflies and humans is crucial to understanding these vectors’ biology, and could be helpful in improving strategies to control leishmaniasis. Several studies investigated compounds that could be attractive to vectors of medical importance, concerning females because of their blood sucking behavior, such as *Anopheles gambiae o*r *Aedes aegypti* [1, 2, 3, 4, 5, 6]. Human skin odors, produced mainly by bacteria from skin microbiota, are known to attract or repulse these insects and, hence, increase or reduce an individual’s risk of infection [2, 5, 7, 8]. Skin local environment can provide greater and specific conditions for microbiota development and establishment. Skin annexes, as hair associated to sebaceous gland, can act also providing this specific environmental for microbiota growth [9] as a retention mesh for volatile compounds that emanate from skin surface, as observed for armpits hair [10]. As a matter of fact, hair has been considered a good matrix for odor profile determination in dogs by using Solid Phase Microextraction in Headspace mode, coupled to Gas Chromatography and Mass Spectrometry (SPME- HS/GC-MS) technique, and it has been considered a potential tool to detect markers for *Leishmania infantum* infection, which can help the diagnosis of visceral leishmaniasis [11, 12].

Despite the fact that there are different methods for human skin odor collection and that there already some very informative studies describing volatiles that compose human skin odors from a diversity of people [13] little is known regarding the behavior of insect vectors of leishmaniasis when exposed to human skin odors. Female *Lutzomyia longipalpis* have been shown to be attracted to human manipulated Petri dishes, but not to unmanipulated ones considered free from human odors [14]. This attraction seems to be mediated by volatile organic compounds (VOCs) from human hands, since a similar effect was obtained using material from manipulated dishes extracted by organic solvents [15]. Additionally, in field testing, *Lutzomyia* (*Nyssomyia) intermedia* and *Lutzomyia* (*Nyssomyia) whitmani* exhibited greater attraction to camping tents with CO_2_ and human bait than to the release of CO_2_ only [16]. In addition, while these studies showed that human odors are attractive to phlebotomine sandflies, the specific attractant volatiles were not identified.

The volatiles present in human skin odors involved in sandfly attraction have not been completely established. *L*. *longipalpis*, but not *L*. (*N*.) *intermedia* nor *Nyssomyia* (*Lutzomyia*) *neivai*, was shown to be attracted by a commercial lure containing lactic acid, caproic acid and ammonia, supposedly mimicking human odor [17, 18]. Experiments performed both in the field and under laboratory conditions demonstrated that phlebotomine sandflies, such as *N*. (*L*.) *neivai* and *L*. (*N*.) *intermedia*, are attracted by (E)-oct-3-en-1-ol (hereafter called octenol) [16, 17, 19, 20], a VOC present in both human breath and sweat [21, 22]. Based on these findings, octenol has been successfully used as a positive control for phlebotomine sandfly attraction in laboratory assays involving *L*. *longipalpis* [19] and *N*. (*L*.) *neivai* [18, 20].

Octenol and nonanol, both of which have been related to human skin odor [23, 24], were demonstrated to attract male and female *L*. *longipalpis* in wind tunnel assays [19]. Female *N*. (*L*.) *neivai* were found to be attracted by octenol, nonanol and decanol, all three VOCs associated with human skin odor [21, 20, 24]. As more than 400 VOCs have already been identified from human skin emanations [13], while very few previously mentioned VOCs have been tested in relation to sandfly vectors, the present study aimed to identify the VOCs from skin odors of individuals residing in an endemic area for American Tegumentary Leishmaniasis (ATL) and evaluate their effects on field-captured female phlebotomine sandfly species in an attempt to search for potential attractants.

## Material and methods

### Study area

Corte de Pedra, located in the Municipality of Tancredo Neves in southeastern Bahia, a State in the northeast of Brazil, is an endemic area for ATL. The Corte de Pedra health clinic is located on the BR-101 highway (13.50’94.80 S, 39.44’84.84 W), 275 km from the city of Salvador, the capital of Bahia [25], and is considered a reference center for the diagnosis and treatment of ATL. More than 90% of the cutaneous leishmaniasis cases (the most common form of ATL) occurring in the region are treated at this clinic, both those originating locally as well as from surrounding areas, including approximately 14 municipalities [26, 27]. *L*. (*N*.) *intermedia* and *L*. (*N*.) *whitmani* have been identified as the main species of vector transmitting *Leishmania* in the region [28, 29].

### Human skin odor sampling

Human skin odors were sampled from 33 healthy male volunteers, aged between 18 and 60 years, all residents of Corte de Pedra. The volunteers were asked to avoid alcohol, not smoke or eat spicy food and to not apply soap when showering, nor use any lotion or perfume (cologne) in the 24 hours prior to sampling. These criteria were adopted to reduce the impact of exogenous sources of odors in the analysis [23, 30].

For human skin odors investigation, at least 60 mg of hair from the legs of volunteers were collected using portable hair clippers, followed by storage at– 20°C until time of analysis. Henceforth, odors or VOCs obtained from hair samples will be referred as odors or VOCs from skin.

### Skin odor analysis

The analysis of skin odors was performed based on a protocol previously described by Oliveira *et al*. [11], with some minor modifications. Three samples of 20 mg of leg hair from each volunteer were collected and sealed in 20 ml glass vials for VOC headspace (HS) microextraction using a Solid Phase Microextraction (SPME) technique. SPME was performed by inserting each vial containing a hair sample in an aluminum heating block, placed upon a temperature controlled plate adjusted to 90°C. A polydimethylsiloxane/divinylbenzene fiber (PDMS/DVB 65 μm, Supelco, Bellefonte) was inserted into the vial via a manual holder through the vial’s silicon septum and exposed for 40 min. After SPME-HS procedures the PDMS/DVB fiber was transported to a gas chromatograph (GC) coupled to a mass spectrometer (MS) and directly inserted into the CG injector of this system for 3 min of thermal desorption. A blank analysis was also performed, by inserting the fiber in an empty vial and following the same procedures described above.

The extracted VOCs were analyzed in the GC-MS system (Shimadzu GC-2010/QP-2010 Plus, Japan) under the following conditions: the DB-1 MS capillary column (30 m x 0.25 μm mm i.d. x 0.25 μm) (Agilent, Palo Alto); He flow rate was 0.7 mL min^-1^; oven temperature program: 40°C for 30 min, 2°C min^-1^ until 130°C, 130°C for 15 min, 2°C min^-1^ until 245°C and 245°C for 4 min; the injector was placed in “splitless” mode at a temperature of 240°C; the temperature of the transfer line was 240°C; the temperature of the ion source was 240°C; the electron impact energy was 70 eV; the scanning frequency was 2 s^−1^ from m/z 40 to m/z 400.

After performing GC-MS procedures, each chromatogram was analyzed with regard to the retention time (RT) of the acquired VOCs. Next, three steps were applied to identify VOCs: (1) Kovats index calculation, based on obtained RTs from the analyzed hair samples and from n-alkanes (C_8_ –C_40_) standard solution (Sigma-Aldrich, USA); (2) identification of compounds by comparison of mass fragmentation patterns with mass spectra contained in the NIST 08 Library; (3) the injection of standard compounds, when available, for RT comparison. The identified VOCs were then searched at PubChem database (https://pubchem.ncbi.nlm.nih.gov/) for Kovats Index comparison aiming to evaluate the reliability of our results. The VOCs detected in blank analysis were excluded from the final list of identified compounds.

### Phlebotomine sandfly capturing and identification

Phlebotomine sandflies were captured using suction light traps, known as Hoover Pugedo (HP) traps [31], in the rural locality denominated “Toca da Onça” (13°32´S; 39°25´W), an area reported to have a high-density sandfly population with a predominance of *L*.*(N*.*) whitmani* and *L*.*(N*.*) intermedia* [28, 29, 32]. The behavior of captured females phlebotomine sandflies were evaluated against human skin VOCs in wind tunnel assays, as it will be explained forward.

Sandflies were maintained in netting cages (15 cm x 15 cm x 15 cm) under 80% relative humidity, an approximate temperature of 26°C and fed with a 50% v/v sucrose solution soaked in thin cotton strips placed above the cages. These conditions were established immediately following capture and maintained during transportation to the laboratory until the time of assay. All evaluated insects were preserved in a 70% alcohol solution immediately after performing the wind tunnel assays. For species identification, each sandfly specimen was slide-mounted with Berlese fluid and morphological characteristics were analyzed in accordance with the taxonomic criteria proposed by Young & Duncan [33]. The replicate was excluded from statistical analysis when any one of the three insects was identified as a different species from *L*. *intermedia* or *L*. *whitmani*, the replicate was excluded from statistical analysis.

### Wind tunnel assays

**Fig 1:**
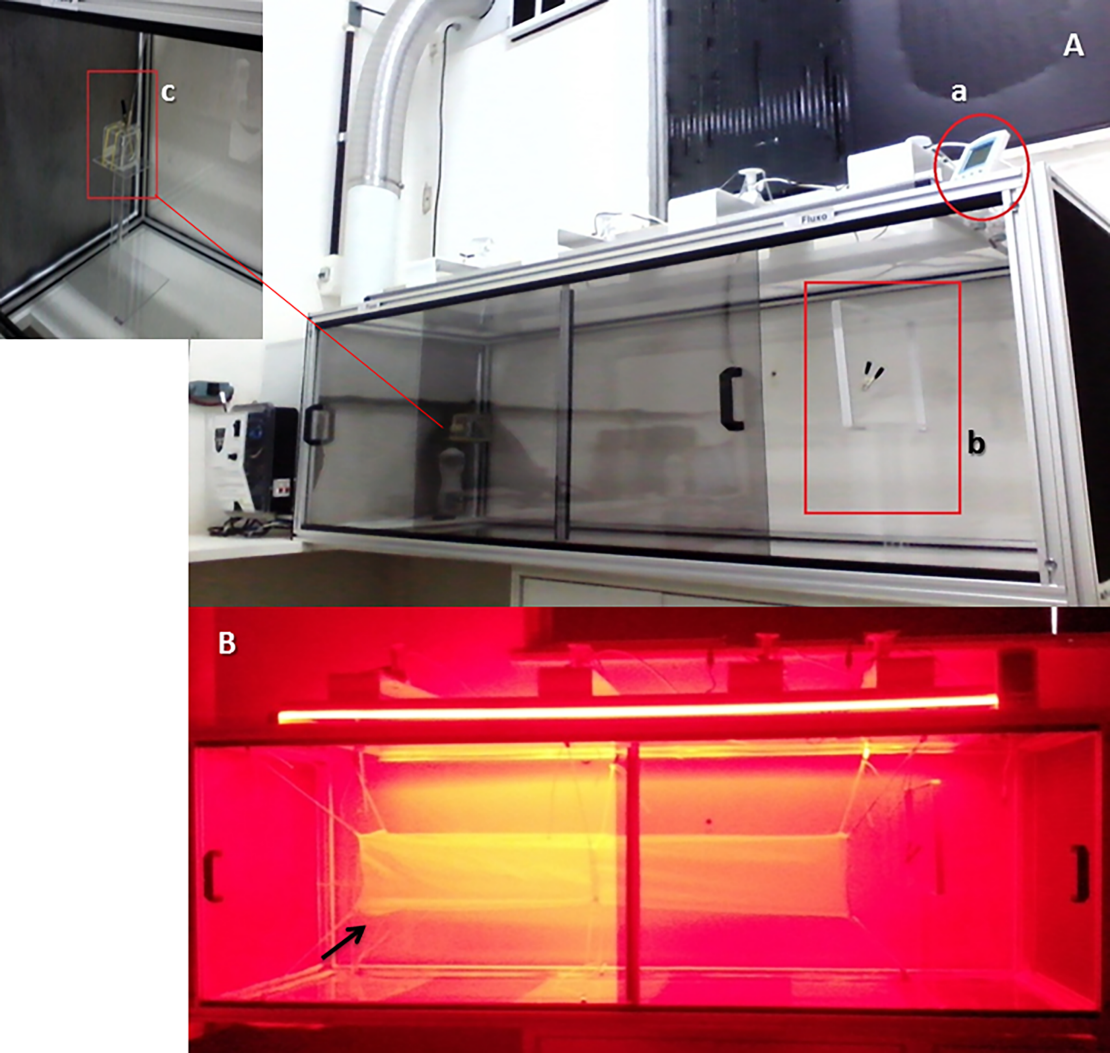
(A) Wind tunnel with a thermohygrometer (a) monitoring temperature and humidity inside the tunnel; (b) Platform to secure the VOC-embedded filter paper; (c) Detail showing the platform with the phlebotomine sandfly release chamber; (B) Wind tunnel under red light illumination with a mini tunnel (black arrow) consisting of white netting.

The wind tunnel assays consisted on activation and attraction tests which experimental details are presented below and were all based on protocols published in other studies employing phlebotomine sandflies in wind tunnel assays [18, 19, 20]. All tests were performed from 09:00 to 19:00 in a transparent acrylic wind tunnel (200 cm x 45 cm x 45 cm) with lateral sliding doors, air flux control (3.06 cm/s) coupled to an exhaust system, at controlled temperatures between 24–26°C, 65–80% humidity and red light to mimic nocturnal illumination (Fig 1). A white netting mini tunnel (120 cm x 15 cm x 15 cm) was placed inside the acrylic wind tunnel to facilitate behavior observation, as well as the recapture of each phlebotomine. Activation was considered when the insect left the releasing chamber and flew until the first third limit of the mini tunnel, while attraction behavior was considered when the insect flew further than the first third limit, i.e. closer to the odor source.

For each test, three female *Lutzomyia* spp. were placed in an acrylic transparent releasing chamber for a 20 min acclimation period in the dark in the absence of food source. The chamber was then positioned on a 25 cm-high platform, facing the mini tunnel with the opening located 110 cm downwind from the odor source (Fig 1). Each tested VOC was delivered pure, if liquid, or dissolved in hexane, if solid (100 mg/ml) via filter paper (4cm x 4 cm), soaked with 200 μL of each compound. The filter paper was positioned above a 25 cm-high platform, facing the opposite end of the netting mini tunnel. At least 10 replicates were conducted for each VOC tested and each experiment lasted 2 min. Hexane was used to clean the wind tunnel after each assay.

Hexane and octenol were used as negative and positive controls, respectively, as proposed in other studies [18, 19, 20]. Seven of the VOCs identified from the human skin, obtained by leg hair samples, Phenylacetaldehyde, 6-methylhept-5-en-2-one, tetradecane, pentadecane, hexadecane, nonadecane and icosane, were evaluated in wind tunnel assays. These VOCs were selected by sorting from a set of twenty three synthetic compounds that we had available for these tests. To validate the wind tunnel system, hexane and octenol were tested prior to experimentation with the other VOCs.

### Chemical compounds

Standard n-alkanes solution and all standard VOCs used in GC-MS analysis and in the wind tunnel assays were at least of 90% purity, and all were purchased from Sigma-Aldrich (USA).

### Statistical analysis

Fisher’s Exact test (F Test) was applied to verify the proportions of activated and attracted insects, always comparing each tested VOC with the negative control (hexane). All analyses were carried out using GraphPad Prism software, version 5.0 (San Diego, CA, USA - www.graphpad.com).

### Ethic statements

This study was approved by the Institutional Review Board of the Gonçalo Moniz Research Institute, Oswaldo Cruz Foundation (protocol number 033030/2015). All volunteers signed a term of free informed consent prior to their participation in this study.

## Results

**Fig 2:**
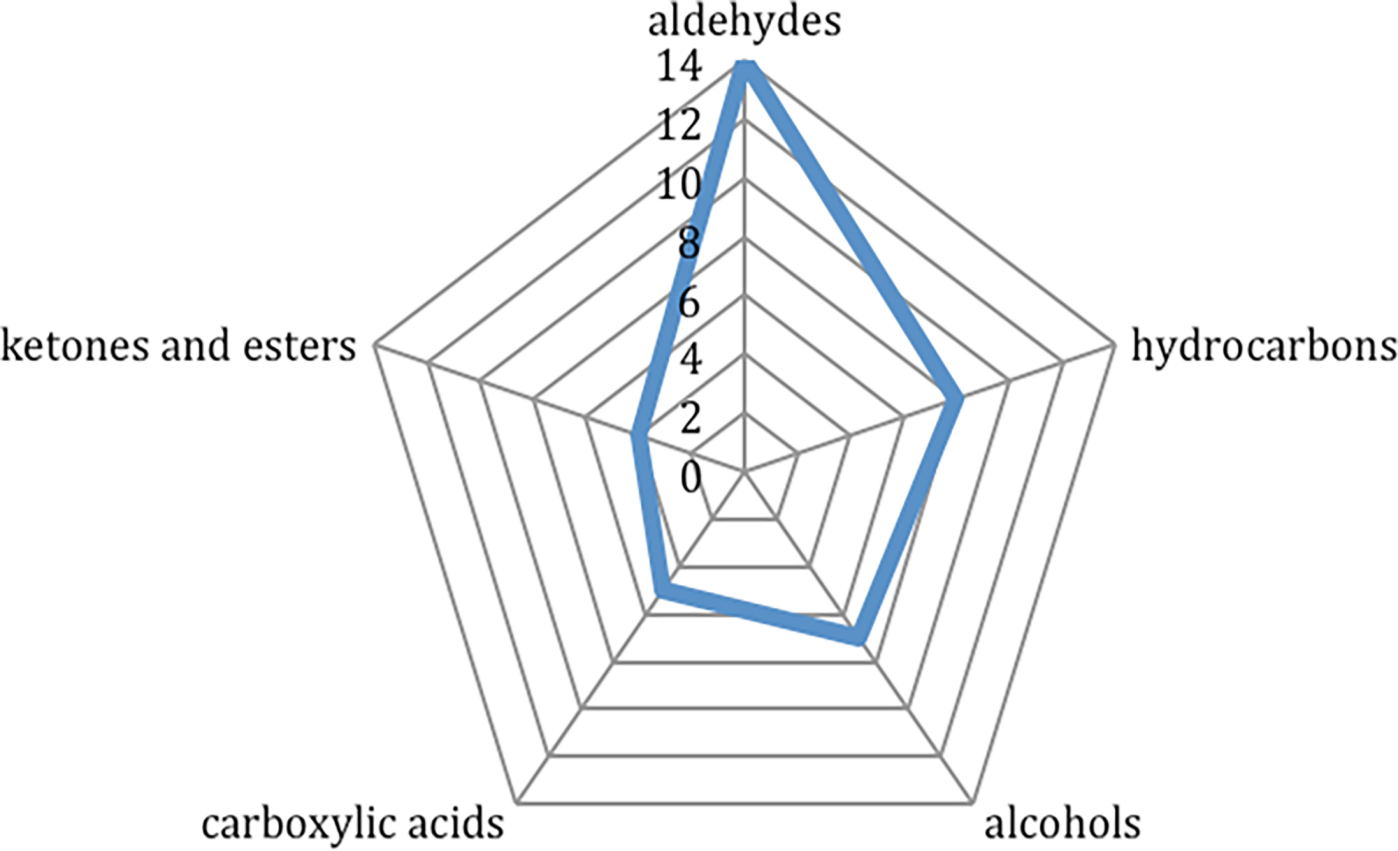
Distribution of the volatile organic compounds identified from the collected human leg hair samples, according to their organic chemical functions.

A total of 42 VOCs were identified from the 99 leg hair samples by HS-SPME/GC-MS analysis (Table 1). All the identified VOCs primarily belonged to six different classes of organic compounds: alcohols, aldehydes, carboxylic acids, ketones, esters and hydrocarbons. Fourteen out of the 42 total identified VOCs (33.3%) were classified as aldehydes, followed by 8/42 (19.1%) as hydrocarbons, 7/42 (16.7%) as alcohols, 5/42 (11.9%) as carboxylic acids. Ketones and esters each represented 4/42 identified VOCs (9.5%) (Fig 2).

**Table 1:** List of the 42 identified volatile organic compounds (VOCs) and each specifically identification method applied.

Compound	RT (min)	Name	%	Functional group	KI_exp_	KI_lit_	CSASI
1	16.10	6-methylhept-5-en-2-one	0.55	Ketone	966	966	X
2	17.15	Octanal	0.51	Aldehyde	981	981	X
3	18.68	2-phenylacetaldehyde	0.04	Aldehyde	1002	1002	X
4	22.42	1-octanol	0.06	Alcohol	1059	1059	X
5	23.74	6-methyl-3,5-heptadien-2-one	0.05	Ketone	1076	1077	
6	24.21	Nonanal	6.59	Aldehyde	1084	1083	X
7	27.00	Cis-verbenol	0.02	Alcohol	1123	1123	
8	27.76	2-nonenal	0.77	Aldehyde	1133	1134	
9	29.50	1-nonanol	0.27	Alcohol	1158	1157	X
10	30.42	Verbenone	0.42	Ketone	1170	1170	X
11	31.36	Decanal	8.90	Aldehyde	1184	1184	X
12	34.85	2-decenal	0.24	Aldehyde	1236	1236	
13	36.91	Nonanoic acid	1.06	Carboxilic acid	1268	1268	X
14	38.25	Undecanal	0.99	Aldehyde	1286	1286	X
15	41.63	2-undecenal	0.49	Aldehyde	1338	1338	
16	44.84	Dodecanal	1.02	Aldehyde	1387	1387	
17	45.75	Tetradecane	0.34	Hydrocarbon	1400	1400	X
18	46.94	Gamma decalactone	0.08	Ester	1414	1414	
19	47.19	Methylparaben	0.31	Ester	1419	1419	
20	47.30	Geranylacetone	2.92	Ketone	1426	1424	
21	48.10	Dodecen-1-al	0.31	Aldehyde	1439	1439	
22	49.32	1-dodecanol	0.66	Alcohol	1456	1455	X
23	51.47	Tridecanal	0.51	Aldehyde	1486	1486	
24	52.44	Pentadecane	1.18	Hydrocarbon	1500	1499	X
25	55.97	(E)-2-tridecenal	0.16	Aldehyde	1541	1537	
26	51.47	Dodecanoic acid	1.67	Carboxilic acid	1554	1554	
27	61.17	Tetradecanal	0.28	Aldehyde	1588	1588	
28	62.55	Hexadecane	1.07	Hydrocarbon	1600	1601	X
29	69.09	1-tetradecanol	6.25	Alcohol	1664	1665	X
30	72.02	Pentadecanal	4.89	Aldehyde	1693	1693	
31	72.90	Heptadecane	1.00	Hydrocarbon	1700	1701	X
32	77.57	Tetradecanoic acid	1.33	Carboxilic acid	1763	1764	X
33	78.32	2-ethylhexyl salicylate	2.26	Ester	1769	1764	X
34	79.20	2-phenyl dodecane	0.16	Hydrocarbon	1791	1791	
35	80.41	Octadecane	1.78	Hydrocarbon	1800	1801	X
36	83.64	Pentadecanoic acid	0.80	Carboxilic acid	1858	1854	
37	84.31	1-hexadecanol	21.13	Alcohol	1866	1865	X
38	86.59	Nonadecane	2.17	Hydrocarbon	1900	1901	X
39	89.56	Hexadecanoic acid	2.57	Carboxilic acid	1957	1956	
40	92.03	Icosane	2.43	Hydrocarbon	2000	2001	X
41	92.62	Isopropil palmitate	8.33	Ester	2013	2013	
42	95.26	1-octadecanol	13.43	Alcohol	2067	2067	X

RT: Retention Time; KI_experimental_: Kovats Index obtained experimentaly; KI_literature_: Kovats Index obtained from literature; CSASI: Confirmed with Synthetic Analytic Standard Injection.

**Fig 3:**
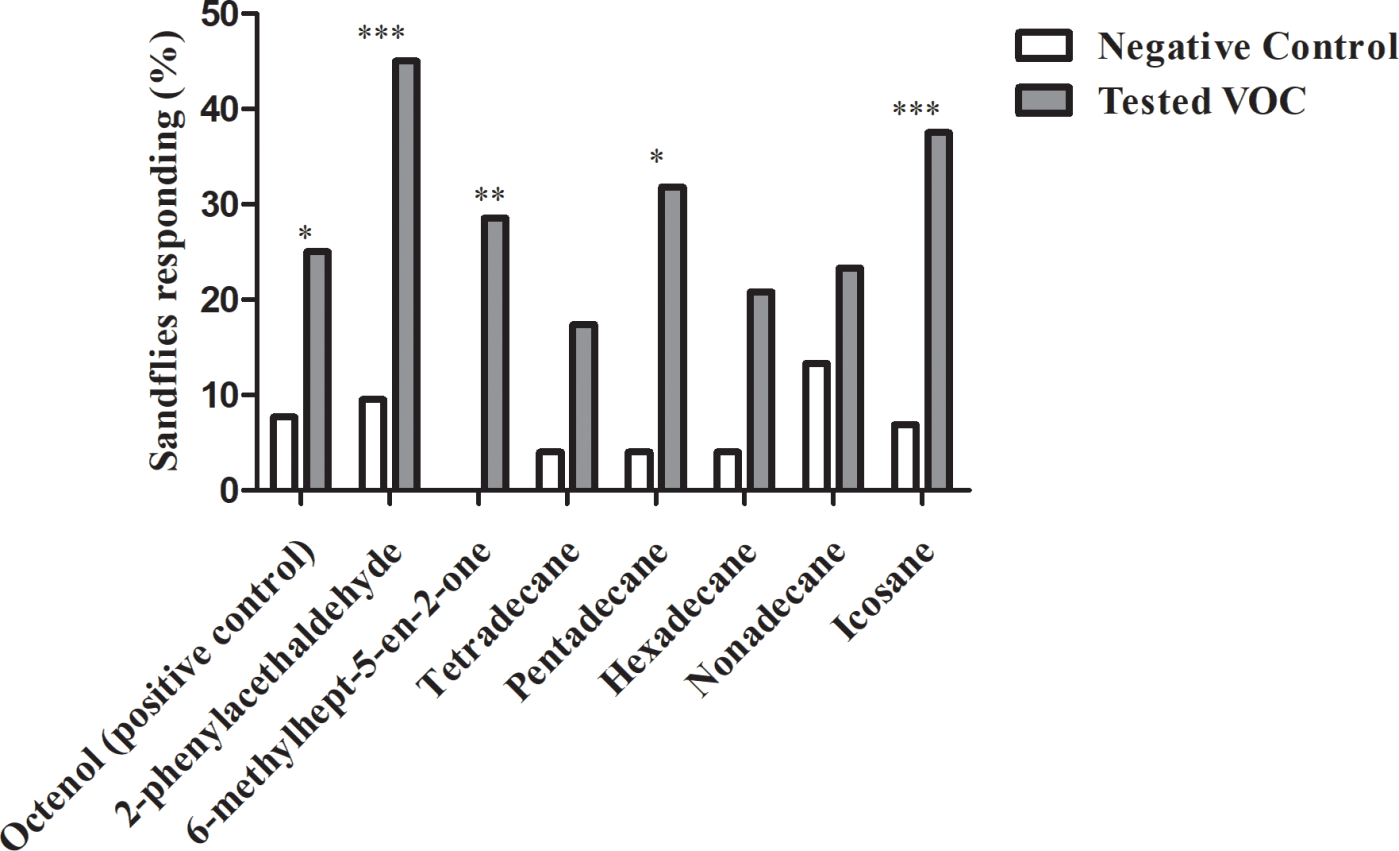
Activation response of captured female phlebotomine sandflies expressed as percentage of activated insects in wind tunnel assays. Hexane was used as a negative control (white bars). * indicates a significant difference in pairwise comparison (p<0.05); ** indicates p<0.01; *** indicates p<0.0001.

**Fig 4:**
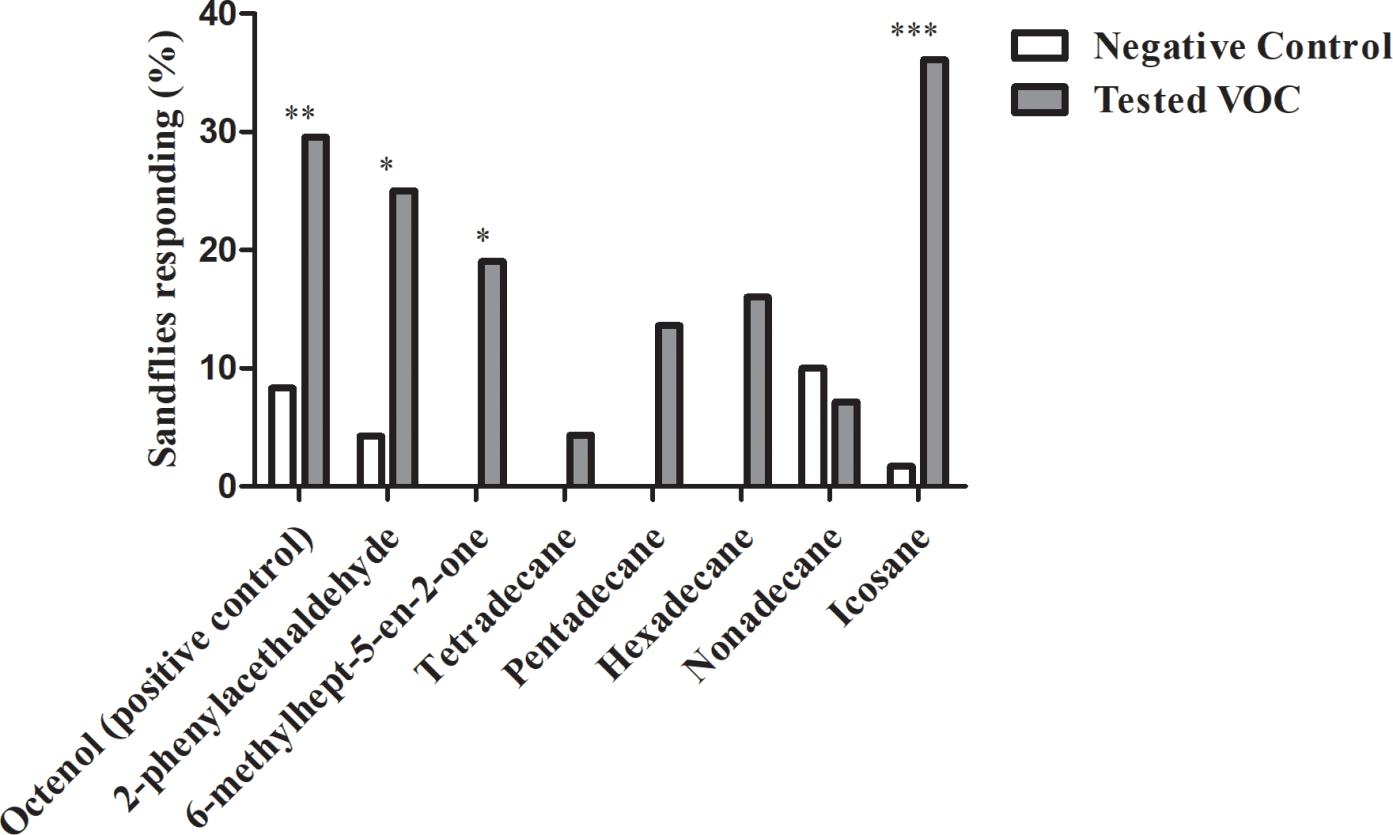
Attraction response of captured female phlebotomine sandflies expressed as percentage of attracted insects in wind tunnel assays. Hexane was used as a negative control (white bars). * indicates a significant difference in pairwise comparison (p<0.05); ** indicates p<0.01; *** indicates p<0.0001.

Validation of the wind tunnel system was performed successfully, as it was observed that octenol activated and attracted a number statistically higher of insects than those observed for hexane (P = 0.0247; P = 0.0076, respectively), confirming what has been described in other wind tunnel assays using *Lutzomyia* species [18, 19, 20]. Four out of the seven tested VOCs induced a significantly higher activation response than the negative control (phenylacetaldehyde P = 0.0004; 6-methylhept-5-en-2-one P = 0.0058; tetradecane P = 0.01800; pentadecane P = 0.0180; hexadecane P = 0.0.0983; nonadecane P = 0.0.5062; icosane P = 0.0.0002 (Fig 3). Phenylacetaldehyde, 6-methylhept-5-en-2-one and icosane were found to attract more female phlebotomine sandflies than the negative control (P = 0.0122, P = 0.0367, P<0.0001, respectively; Fig 4).

Post-assay phlebotomine sandfly identification was successfully performed on 83.8% of the 420 specimens used in wind tunnel assays, with a predominance of *Lutzomyia intermedia* (75.4%) followed by *Lutzomyia migonei* (4.5%), *Luztomyia whitmani* (2.8%), *Lutzomyia fischeri* (0.9%), *Lutzomyia shannoni* (0.2%). The other 16.2% of the insects was not identified due to damage during handling. Statistical analysis indicated that the phlebotomine sandflies used in each of the wind tunnel assays were similar with regard to species, with *Lutzomyia intermedia* being the most predominant.

## Discussion

The present study employed human leg hair samples to identify which VOCs emanating from human skin surface are potentially attractive to anthropophilic phlebotomine sandfly species. We also aimed to evaluate the attraction capability of some of the identified VOCs against the field-captured female sandflies in laboratory wind tunnel assays. The species of phlebotomine sandflies is not always well known in field applications, and rarely is a unique species representative of a given area; nonetheless, one species may predominate in a specific area. It follows that the attraction of phlebotomine sandflies to human odors represents a potential risk for leishmaniasis transmission. The present study approximated natural conditions in a laboratory setting to evaluate the attraction of field-captured sandflies to volatiles arising from skin odors identified in individuals residing in the same area of insect collection. So far, we have found one aldehyde and one hydrocarbon that may be suitable for further testes and may be involved in attraction of anthropophilic phlebotomine sand flies species.

Our investigation of the skin odor profile from individuals residing in the study area resulted in 42 VOCs, some of which could represent a potential tool for supporting strategies capable of avoid human exposure to sandflies bites. In addition to human skin, some of these volatiles had already been identified in plants, which could represent glucose feeding sources for male and female phlebotomine sandflies [34]. Phenylacetaldehyde, for example, was shown to induce activation and attraction behavior in *Lutzomyia intermedia*. This compound, which is also present in flower odor of *Silene otites* (Caryophyllaceae), was demonstrated to induce electroantennographic activity in *Culex pipiens* mosquitos, a vector of diseases such as West Nile virus and Japanese encephalitis, and was further shown to be attractive to these insects in wind tunnel assays [35].

In the present study, 6-methylhept-5-en-2-one was also found to activate and attract significantly more phlebotomine sandflies than the negative control. Ketone 6-methylhept-5-en-2-one has been reported as a potential repellent of *Anopheles gambiae*, *Aedes aegypti* and *Culex quinquefasciatus* [3, 36]. However, while these mosquito species are capable of detecting this compound, its repellent capability remains unclear, as it actually could simply mask chemical cues that attract these mosquitoes during host seeking [3, 37].

According to our results 6-methylhep-5-en-2-one is an attractive human volatile to phlebotomine sandflies, differently from what has already been shown to other insects of medical importance, as *Anopheles gambiae*, *Culex quinquefasciatus* and *Aedes aegypti* [3, 36]. Sulcatone was tested also against non-hematophagous dipterans and reported as an attractant to *Musca autumnalis* (Diptera: Muscidae) in wind tunnels assays [38]. Interestingly, sulcatone is referred as an important cue that helps hematophagous insects to distinguish humans from other hosts [39] and somehow such VOC can in fact be considered an attractant. According to McBride [39], this paradox can be better understood if we consider that sulcatone behavior effects can depend on context or on concentration, in a way that small concentrations, for example, especially in a blend of other human VOCs, can evoke an attraction behavior upon mosquitoes.

Despite the fact that several studies have reported that 6-methylhept-5-en-2-one as repulsive for mosquitoes [3, 36, 40] the effects of this VOC on other hematophagous insects is still little known. Thus, our results can be an important contribution to the understanding of the effects of this ketone, which is so characteristic in human skin odors profiles and that also seems to be a well recognizing cur to hematophagous insects.

All tested hydrocarbons, tetradecane (C_14_), pentadecane (C_15_), hexadecane (C_16_), nonadecane (C_19_) and icosane (C_20_), induced a higher total number of activated and attracted insects than the negative control. However, only pentadecane and icosane were found to induce significantly greater activation responses, while icosane was shown to attract significantly more phlebotomine sandflies than controls. No previous studies have reported any biological functions, e.g. attraction or repulsion, exercised by these hydrocarbons in insects of medical importance. Accordingly, this is the first report indicating that pentadecane and icosane activated flight behavior and, as such, are probably recognized by ATL vectors; i.e. icosane is an attractant for these insects.

It is important to observe that the previous studies performed in wind tunnel assays using phlebotomine sandflies obtained higher proportions of attraction responses of these insects [18, 19, 20]. For octenol (100%), for example, these studies obtained responses rates up from 60%, therefore higher than rates obtained in our observations. However, these researches were performed using slightly different methods from those applied here, by using, for example, laboratory reared phlebotomine sandflies, instead of field captured ones [19, 20], fact that make feasible to know the age of all insects used in the experiments, which is not possible using field captured insects. An exception is the research performed by Pinto *et al* [18], neverless this and all other wind tunnel studies evaluated species different from *Lutzomyia (Nyssomyia) intermedia*.

Among these considerations, we are convinced that once the tested compounds elicited significantly higher responses than the negative control they may be considered as attractants, no matter the obtained percentage responses. It is also crucial to emphasize that all VOCs we tested were present in human skin odors, which clearly support the idea that they can be effective in attract anthropophilic insects species. Although we identified 42 VOCs in human skin odor collected from individuals residing in an area endemic for ATL, we were only able to test seven of these. Regrettably, the field capture of phlebotomine sandflies is a daunting task, and captures often do not return a sufficient quantity of insects to adequately conduct testing. Furthermore, at the time of wind tunnel assaying, we had not finished the quantitative analysis and thus we selected these seven compounds randomly, by sorting.

Accordingly, we must emphasize that more extensive testing should be performed to more comprehensively elucidate the effects of the other VOCs, in addition to any possible combinations. Farther, it should be considered that the list of VOCs obtained from people that live in an endemic area for ATL can be largely used in other studies, including human skin odor profile comparisons (as frequently this kind of study refers to people that are not from risk areas for vector borne diseases) and searching of specific VOCs that can elicit any behavior response to phlebotomine sandflies even under laboratory as field conditions.

## Conclusions

The present results corroborate the notion that ATL vectors are attracted to volatile organic compounds present in odors arising from human skin, which could represent candidates for use in the field to enhance the capture efficiency of conventional traps, thereby improving the monitoring and control of anthropophilic phlebotomine sandflies. In addition, better traps could also assist in the collection of specimens for laboratory studies on the prevention and control of leishmaniasis. Our initial results suggest that 6-methylhept-5-en-2-one, 2-phenylacetaldehyde and icosane may be suitable candidates for attractiveness experimentation in the field to enhance the capture efficiency of phlebotomine sandflies, notably *Lutzomyia intermedia*, using capture traps with VOC dispensers.

Additional investigation must be conducted to elucidate the main VOCs present in human skin odors that serve to attract anthropophilic phlebotomine sandflies. New sandfly captures in endemic areas will be necessary to test the other VOCs that were identified in this study. Moreover, in accordance with our findings, we suggest that further testing involving 6-methylhept-5-en-2-one, 2-phenylacetaldehyde and icosane be conducted in the field to more comprehensively evaluate the effects of these VOCs on the capture efficiency of phlebotomine sandflies.
